# MiR-1180 promotes apoptotic resistance to human hepatocellular carcinoma via activation of NF-κB signaling pathway

**DOI:** 10.1038/srep22328

**Published:** 2016-03-01

**Authors:** Guosheng Tan, Linwei Wu, Jinfu Tan, Bing Zhang, William Chi-shing Tai, Shiqiu Xiong, Wei Chen, Jianyong Yang, Heping Li

**Affiliations:** 1 Department of Interventional Radiology, the First Affiliated Hospital of Sun Yat-sen University, Guangzhou 510080, P.R. China; 2 Organ Transplantation Center, the First Affiliated Hospital, Sun Yat-Sen University, Guangzhou, China; 3 Department of Anorectal & Hernial Surgery, the First Affiliated Hospital of Sun Yat-sen University, Guangzhou 510080, P.R. China; 4 Department of Nuclear Medicine, the First Affiliated Hospital of Sun Yat-sen University, Guangzhou 510080, P.R. China; 5 Centre for Cancer and Inflammation Research, School of Chinese Medicine, Hong Kong Baptist University. Kowloon Tong, Hong Kong, S.A.R., P.R. China; 6 Institute of Integrated Bioinformedicine and Translational Science, Hong Kong Baptist University, Shenzhen Research Institute and Continuing Education, Shenzhen 518000, P.R. China; 7 Department of Biochemistry, University of Leicester, Leicester, UK; 8 Department of Medical Oncology, the First Affiliated Hospital of Sun Yat-sen University. Guangzhou 510080, P.R. China

## Abstract

Apoptosis resistance in human hepatocellular carcinoma (HCC) is a significant factor in carcinogenesis. Therefore, understanding the molecular mechanisms involved in apoptosis resistance is crucial for developing anticancer therapies. Importantly, small non-coding microRNAs (miRNAs) have been reported as key biomarkers for detecting tumour onset and progression. In the present study, we demonstrate that miR-1180 is upregulated in HCC. Ectopic expression of miR-1180 has an anti-apoptotic effect in HCC, while miR-1180 inhibition increases cell apoptosis, both *in vitro* and *in vivo*. Moreover, our results show that miR-1180 directly targets key inhibitors of the nuclear factor (NF)-κB signaling pathway (i.e., OTUD7B and TNIP2) and the pro-apoptotic Bcl-2 associated death promoter (BAD) protein by post-transcriptional downregulation. Therefore, the anti-apoptotic function of miR-1180 in HCC may occur through NF-κB pathway activation via downregulation of its negative regulators. In conclusion, our study reveals the critical role of miR-1180 during apoptosis resistance in HCC.

Human hepatocellular carcinoma (HCC) is the fifth most frequent malignancy and the third most common cause for cancer mortality worldwide. The incidence of HCC is particularly high in Asia, where the high prevalence of hepatitis B and hepatitis C strongly predisposes individuals to the development of chronic liver disease, and subsequently, HCC^1^,^2^. There are no obvious symptoms during the early stages of disease; HCC patients are commonly diagnosed at an advanced or unresectable stage^3^,^4^. Apoptosis resistance in HCC is a significant factor for carcinogenesis and tumour progression, and is the major cause of drug resistance leading to therapy failure^5^,^6^. Therefore, a greater understanding of the molecular mechanisms involved in apoptosis resistance is crucial for developing novel anticancer strategies and improving therapeutic efficacy.

This study investigated the molecular mechanisms of a microRNA (miRNA) in apoptosis resistance. MiRNAs are evolutionarily conserved, non-coding RNAs involved in several biological processes, such as proliferation and apoptosis^7^,^8^. Several studies have demonstrated their role in tumour onset and progression, and their use as novel biomarkers for subsequent diagnosis and therapy^9^,^10^,^11^,^12^,^13^,^14^,^15^. These miRNAs control gene expression either by degradation of target mRNAs or post-transcriptional repression through targeting the 3′-untranslated region (3′-UTR) in a sequence-specific manner^8^,^9^. Notably, multiple genes, that comprise survival pathway components and contribute to apoptosis resistance, have been identified as potential targets of several miRNAs^16^,^17^,^18^. Hence, tumour-associated miRNAs may represent a novel group of viable targets for therapeutic intervention.

In the present study, we demonstrate the upregulation of miR-1180 in HCC, which is further supported by the published micro-array-based high-throughput assessment (NCBI/GEO/GSE36915). We find that the ectopic expression of miR-1180 has an anti-apoptotic effect in HCC, while its inhibition increases cell apoptosis. Moreover, we reveal that the miR-1180 directly targets two negative regulators of the nuclear factor (NF)-κB signaling pathway (OTUD7B and TNIP2)^19^,^20^,^21^, as well as the pro-apoptotic Bcl-2 associated death promoter (BAD) protein, via post-transcriptional downregulation. Therefore, the anti-apoptotic function of miR-1180 in HCC may be induced via NF-κB activation by downregulation of NF-κB-negative regulators.

## Materials and Methods

### Ethics statement

All samples were obtained with informed consent and approved by the Institutional Research Ethics Committee. Informed consent was obtained from all participants involved in the study. All the experiments were carried out in accordance with the approved guidelines of the Institutional Research Ethics Committee of Sun Yat-Sen University.

### Cell culture and treatment

Immortalized normal liver epithelial cells (THLE3) and HCC cell lines (Hep3B, HepG2, Bel-7402, HCCC-9810, MHCC97H, MHCC97L, Huh7 and QGY-7703) were purchased from the American Type Culture Collection (ATCC, Manassas, VA, USA). All cells were cultured under the conditions described by the manufacturers’ instruction and were grown in RPMI-1640 (Invitrogen, Carlsbad, CA, USA) supplemented with 10% fetal bovine serum (Invitrogen). The miR-1180 mimic, miR-1180 inhibitor, negative control (NC), antagomiR-1180 and antagomiR control were purchased from RiboBio Co. Ltd (Guangzhou, Guangdong, China). Transfection of oligonucleotides was performed using the Lipofectamine 2000 reagent (Invitrogen), according to the manufacturers’ protocol. Cisplatin (Sigma, Saint Louis, MO, USA), dissolved in 1× PBS, was used as the apoptosis inducer (0.4 μg/ml, 24 h) and JSH23 (Sigma; dissolved in 10 μΜ with DMSO, 24 h) was used as the NF-κB inhibitor.

### Tissue specimens

Fresh HCC tissues and normal hepatic tissues were collected from curative resection and diagnosed histopathologically at the Department of Interventional Radiology, the First Affiliated Hospital of Sun Yat-Sen University. The samples were immediately frozen and stored in liquid nitrogen (–80 °C) after surgery prior to analysis. Informed consent was obtained from all participants, and the study was approved by the Institutional Research Ethics Committee.

### Cell viability assay

Cell proliferation was determined by the MTS [3-(4, 5-dimethylthiazol-2-yl)-5-(3- carboxymethoxyphenyl)-2-(4-sulfophenyl)-2H-tetrazolium, inner salt] (CellTiter96; Promega) assay. Cells were plated into 96-well plates (3000 cells per well) and incubated with MTS at 37 °C for 4 h. The optical density (OD) at 490 nm was determined with a microplate reader (Bio-Tek EL-311; BioTek Instruments Inc., Winooski, USA). Results are representative of three independent experiments with triplicate samples for each cell line.

### Colony formation assay

Cells were plated into a 6-well plate (1 × 10^4^ cells per well) and transfected with miR-1180 mimic, miR-1180 inhibitor, or NC. After 24 h, the cells were treated with cisplatin (0.4 μg/ml) for another 24 h, and then cultured for 10 days. The colonies were stained with 1.0% crystal violet for 5 min after fixation with 10% formaldehyde for 15 min. All experiments were performed in triplicate.

### Flow cytometry analysis

For cell cycle analysis, the cells were harvested and fixed in a solution of 70% ice-cold ethanol with phosphate buffered saline (PBS). Propidium iodide (PI, 50 μg/ml; Sigma) and a fluorescein isothiocyanate (FITC)-conjugated monoclonal antibody specific for Annexin V (Sigma) were incubated with the cells at 4 °C for 30 min before analysis using the FACS Calibur system (BD Biosciences, CA, USA). The data were analysed by the ModFit LT software package. Results are representative of three independent experiments with triplicate samples for each cell line.

### Xenografted tumour model and treatment

BALB/c-nu mice (5–6 weeks old, 18–20 g) were purchased from the Experimental Animal Center of the Guangzhou University of Chinese Medicine and housed in barrier facilities on a 12 h light/dark cycle. The Institutional Animal Care and Use Committee of Sun Yat-Sen University approved all experimental procedures. The mice were randomly assigned to groups (n = 5/group). All mice in groups were inoculated subcutaneously with HepG2 cells (1 × 10^7^ cells per mouse) in the left dorsal flank. A week later, mice in group A were injected intratumourally with 100 μl of a miR-1180 antagonist (diluted in PBS at 2 mg/ml), and mice in group B were injected with a antagomiR control (diluted in PBS at 2 mg/ml), three times per week. After two weeks, all the mice were injected intratumourally with cisplatin (0.4 μg/ml) three times per week. Tumours were examined twice weekly; length, width, and thickness were measured with callipers, and tumour volumes were calculated. Tumour volume was calculated using the equation (L × W^2^)/2. On day 40, the animals were euthanised, and the tumours were excised and weighed.

### Western blotting assay

Total protein was extracted from whole cells. The samples were heated at 90 °C for 10 min with the 1× sample buffer and separated by SDS-PAGE. Subsequently, the sample was electroblotted onto a polyvinylidene difluoride (PVDF) membrane (Millipore, Billerica, MA, USA). The membranes were probed with polyclonal rabbit antibodies against anti-Bcl-2, anti-Bax, anti-cleaved-caspase-3, anti-OTUD7B, anti-TNIP2, and anti-BAD (Abcam, Cambridge, MA, USA). For loading control, the membranes were stripped and re-probed with anti-alpha-Tubulin (Abcam).

### Luciferase reporter assay

Cells were seeded in triplicate into a 24-well plate (3 × 10^4^ per well) and were allowed to settle for 12 h. The pNF-κB-luciferase plasmid (100 ng) or the control-luciferase plasmid, and an additional 10 ng of pRL-TK Renilla plasmid (Promega, Madison, WI, USA), were transfected into HCC cells using the Lipofectamine 2000 reagent (Invitrogen). Luciferase and Renilla signals were measured at 48 h, post-transfection using the Dual Luciferase Reporter Assay Kit (Promega), according to a protocol provided by the manufacturer.

### RNA extraction and quantitative reverse-transcription PCR (qRT-PCR)

Total cellular RNA was extracted using Trizol reagent (Invitrogen) according to a protocol provided by the manufacturer. The SYBR Green I (Molecular Probes, Invitrogen) dye was used according to the manufacturer’s instructions, and qRT-PCR was performed and analysed using a 7500 Fast Real-Time Sequence detection system software (Applied Biosystems, Foster City, CA, USA). The qRT-PCR reactions for miRNAs were performed at 95 °C for 3 min, followed by 40 cycles (each 30 s in length) at 95 °C, 58 °C, and 72 °C. The expression levels of miR-1180 were normalized with reference to the expression levels of U6 small nuclear RNA (snRNA). The qRT-PCR conditions for genes were set at 95 °C for 10 min, followed by 40 cycles at 95 °C for 20 s, 60 °C for 30 s and 72 °C for 1 min. Glyceraldehyde 3-phosphate dehydrogenase (GAPDH) was used as a reference gene that acts as an internal standard to normalize the mRNA expression. The fold changes were calculated by relative quantification (2^−ΔΔCt^). The primers used for stem-loop reverse-transcription PCR of miR-1180 and U6, were purchased from RiboBio (RiboBio Co. Ltd, Guangzhou, Guangdong, China). Other primers used included: *CCND1* forward: 5′-AACTACCTGGACCGCTTCCT-3′, *CCND1* reverse: 5′- CCACTTGAGCTTGTTCACCA-3′; *IL6* forward: 5′-TCTCCACAAGCGCCTTCG-3′, *IL6* reverse: 5′-CTCAGGGCTGAGATGCCG-3′; *MYC* forward: 5′-TCAAGAGGCGAACACACAAC-3′, *MYC* reverse: 5′-GGCCTTTTCATTGTTTTCCA-3′; *Bcl-XL* forward: 5′-TCCTTGTCTACGCTTTCCACG-3′, *Bcl-XL* reverse: 5′-GGTCGCATTGTGGCCTTT-3′; *TNFA* forward: 5′-CCAGGCAGTCAGATCATCTTCTC-3′, *TNFA* reverse: 5′-AGCTGGTTATCTCTCAGCTCCAC-3′; *VEGF* forward: 5′-GTGTCCAGTGTAGATGAACTC-3′, *VEGF* reverse: 5′-ATCTGTAGACGGACACACATG-3′; *GAPDH* forward: 5′-GACTCATGACCACAGTCCATGC-3′, *GAPDH* reverse: 3′-AGAGGCAGGGATGATGTTCTG-5′.

### Electrophoretic Mobility Shift Assay (EMSA)

Electrophoretic mobility shift assay was performed by using the LightShift Chemiluminescent EMSA kit (Thermo Fisher Scientific, Waltham, MA, USA), according to the manufacturer’s instructions. The nuclear protein exaction was performed by using the NE-PER™ Nuclear and Cytoplasmic Extraction Kit (Thermo Fisher Scientific). DNA probes containing specific binding sites were used as described previously^22^ (NF-κB sense, 5′-AGTTGAGGGGACTTTCCC AGGC-3′, antisense, 5′-GCCTGGGAAAGTCCCCTCAAC-3′; OCT-1 sense, 5′-TGTCGAATGCAAATCACTAGAA-3′, antisense, 5′-TTCTAGTGATTTGCATTCGAC A-3′).

### Statistical analyses

All quantitative data were analysed using a two-tailed paired Student’s *t*-test. The data were expressed as the mean ± standard deviation (SD) for three independent experiments. Survival curves were plotted by the Kaplan-Meier method and compared by the log-rank test. *P* < 0.05 was considered to indicate a statistically significant difference.

## Results

### MiR-1180 is upregulated in HCC

**Figure 1:**
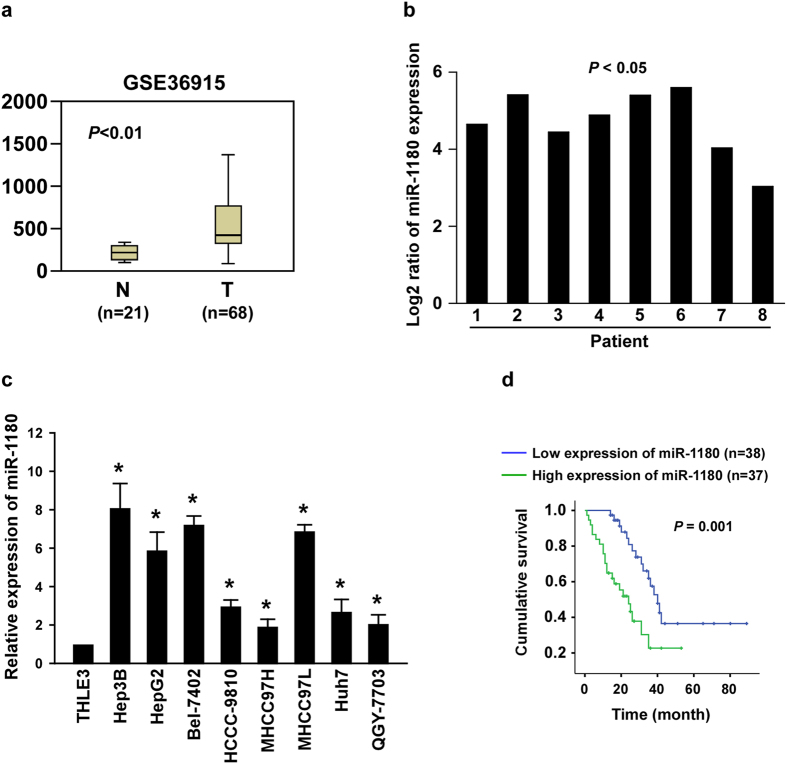
miR-1180 is upregulated in HCC. (**a)** The expression of miR-1180 in HCC tissues (T) compared to normal hepatic tissues (N), as measured using a published micro-array-based high-throughput assessment (*P* < 0.05; NCBI/GEO/GSE36915). **(b**) qRT-PCR analysis of miR-1180 expression in eight cancerous tissues (T) paired with their adjacent noncancerous hepatic tissues (ANT); Log2 value was used to show the expression difference. (**c)** qRT-PCR analysis of miR-1180 expression in a panel of hepatocellular carcinoma cell lines compared to normal liver epithelial (THLE3) cells. (**d)** Kaplan-Meier curves of HCC patients with low- versus high-expression of miR-1180 (n = 75; *P* = 0.001, log-rank test). The average miR-1180 expression was normalized using U6 expression. Each bar represents the mean ± SD of three independent experiments. *P < 0.05.

By analysing a published micro-array-based high-throughput assessment (NCBI/GEO/GSE36915), we found that miR-1180 was markedly upregulated in HCC tissues (T, n = 68), compared with that in non-tumour tissues (N, n = 21; *P* < 0.01; Fig. 1a). To confirm the result of the published assessment, we examined the expression level of miR-1180 in our freshly collected HCC tissues using qRT-PCR. We found miR-1180 was upregulated in eight samples of HCC tissues (T) compared with their adjacent noncancerous hepatic tissues (ANT; Fig. 1b). The expression of miR-1180 was also significantly increased (*P* < 0.05) across a panel of HCC cell lines (i.e., Hep3B, HepG2, BEL-7402, HCCC-9810, MHCC97H, MHCC97L, Huh7, and QGY-7703) compared to normal liver epithelial cells (THLE3; Fig. 1c). As miR-1180 expression was elevated in HCC tissues and cell lines, miR-1180 appears to have a positive function on HCC progression. In addition, we found that HCC patients with higher miR-1180 expression had a shorter survival time, whereas patients with lower miR-1180 expression had a longer survival time (n = 75; *P* = 0.001; Fig. 1d), suggesting a potential correlation between expression of miR-1180 and the progression of HCC.

### Ectopic miR-1180 promotes proliferation and inhibits cisplatin-induced apoptosis of HCC cells

**Figure 2:**
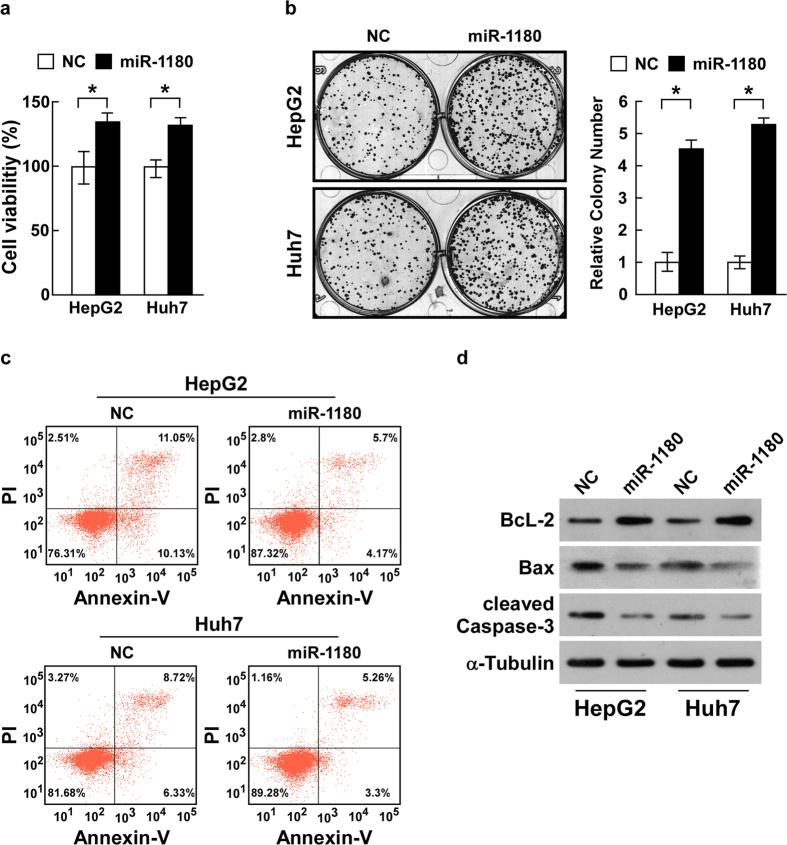
Ectopic miR-1180 promotes cell viability and inhibits cisplatin-induced apoptosis of HCC cells. **(a)** Ectopic miR-1180 promotes cell viability in HepG2 and Huh7 cell lines compared with a negative control (NC) as measured by the MTS assay. (**b)** Representative micrographs (left) and quantifications (right) of crystal violet stained cell colonies formed by HepG2 and Huh7 cell lines, 10 days after inoculation. (**c)** Flow cytometry experiments using Annexin V/PI double staining to determine the percentages of apoptotic cells in HepG2 and Huh7 cell lines. (**d)** Western blotting analysis of Bcl-2, Bax and cleaved-caspase-3 expression in miR-1180 overexpressing cells compared with a negative control (NC); α-Tubulin is the loading control. Each bar represents the mean ± SD of three independent experiments. *P < 0.05.

To further explore the role of miR-1180 upregulation in HCC progression, we chose to examine the HepG2 and Huh7 cell lines (Supplementary Figure 1a). We first monitored the impact of ectopic overexpression of miR-1180 on cell viability using the MTS assay. Ectopic miR-1180 significantly increased cell viability compared to the control cells (*P* < 0.05; Fig. 2a). Our colony formation assays showed that overexpression of miR-1180 led to more and larger-sized colonies compared to control cells (*P* < 0.05; Fig. 2b). To further explore the function of miR-1180 on cytotoxic reagent-induced cell apoptosis, HCC cells were exposed to an anti-tumour agent, cisplatin. Flow cytometry experiments demonstrated that, after treatment with cisplatin (0.4 μg/ml, 24 h), the percentage of Annexin-V-FITC-positive HCC cells in cells that were overexpressing miR-1180 decreased significantly compared to the control cells (Fig. 2c). We also examined the expression of the anti-apoptotic protein, Bcl-2, and the pro-apoptotic proteins, Bax and caspase-3. Overexpression of miR-1180 resulted in an increase of Bcl-2 expression, and a decrease of Bax and cleaved-caspase-3 expression (Fig. 2d, Supplementary Figure 2a). Together, these results indicate that overexpression of miR-1180 in HepG2 and Huh7 cell lines can promote cell proliferation and provide resistance to cisplatin-induced cell death.

### Inhibition of miR-1180 attenuates cell viability and promotes cisplatin-induced apoptosis of HCC cells *in vitro*

**Figure 3:**
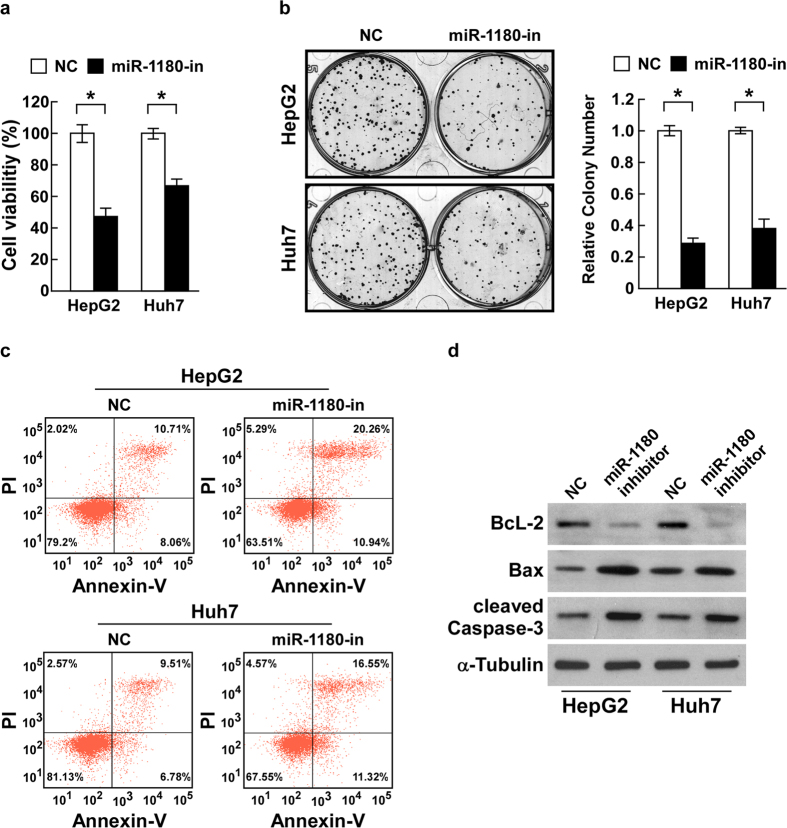
Inhibition of miR-1180 suppresses cell viability and promotes cisplatin-induced apoptosis of HCC cells *in vitro*. **(a)** Inhibition of miR-1180 (with the miR-1180-in) decreases cell viability in HepG2 and Huh7 cell lines compared with a negative control (NC) as measured by the MTS assay. (**b)** Representative micrographs (left) and quantifications (right) of crystal violet stained cell colonies formed by HepG2 and Huh7 cell lines, 10 days after inoculation. **(c)** Flow cytometry experiments using Annexin V/PI double staining to determine the percentages of apoptotic cells in HepG2 and Huh7 cell lines. **(d)** Western blotting analysis of Bcl-2, Bax, and cleaved-caspase-3 expression in miR-1180 overexpressing cells compared to control cells; α-Tubulin is the loading control. Each bar represents the mean ± SD of three independent experiments. *P < 0.05.

Suppression of miR-1180 (loss-of-function) studies using an inhibitor was performed to confirm the function of miR-1180 in HCC cells (Supplementary Figure 1b). The suppression of miR-1180 caused a significantly decreased cell viability compared to the control cells (*P* < 0.05; Fig. 3a). Colony formation assays showed that cells overexpressing miR-1180 showed fewer and smaller-sized colonies than control cells (Fig. 3b). Concurrently, apoptosis analysis using flow cytometry showed an increase in the percentage of Annexin-V-FITC-positive in cisplatin-treated and miR-1180-inhibited HCC cells compared to the control cells (Fig. 3c). Furthermore, inhibition of miR-1180 led to a decrease in Bcl-2 expression and an increase in Bax and cleaved-caspase-3 expression (Fig. 3d, Supplementary Figure 2b). Moreover, the ratio of Bcl-2/Bax was significantly increased by miR-1180 overexpression and decreased by miR-1180 suppression, suggesting that miR-1180 increases the anti-apoptotic ability of HCC (Supplementary Figure 2c). These results strongly suggest that inhibition of miR-1180 attenuates cell viability and promotes cisplatin-induced apoptosis of HCC cells.

### Inhibition of miR-1180 suppresses HCC cells proliferation and promotes cisplatin-induced apoptosis *in vivo*

**Figure 4:**
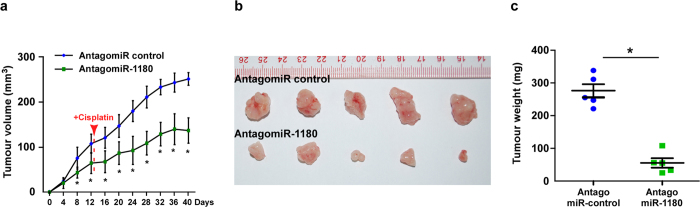
Inhibition of miR-1180 with a miR-1180 antagonist (AntagomiR-1180) suppresses tumour growth and promotes cisplatin-induced apoptosis of HCC cells *in vivo*. (**a**) Tumour volumes were measured on the indicated days and presented as the mean ± SD. Red arrow indicates the first time point of cisplatin treatment. (**b**) Images of tumours from all mice in each group. (**c**) Tumour weights (mg) in each group. *P < 0.05.

We then examined the tumour suppressive role of a miR-1180 antagonist in HCC progression using an *in vivo* tumour model. Importantly, intratumoural injection with an miR-1180 antagonist dramatically inhibited tumour growth, while injecting a antago-miR control had no effect on tumour development (Fig. 4a). Moreover, after the tumours injected with cisplatin, tumours treated with the miR-1180 antagonist were significantly decreased in both size and weight compared to those tumours injected with the antago-miR control (Fig. 4a–c). Collectively, these results suggest that the inhibition of miR-1180 suppresses HCC cells proliferation and promotes cisplatin-induced apoptosis *in vivo*.

### miR-1180 modulates HCC cells survival through downregulation of its target genes, OTUD7B, TNIP2 and BAD

**Figure 5:**
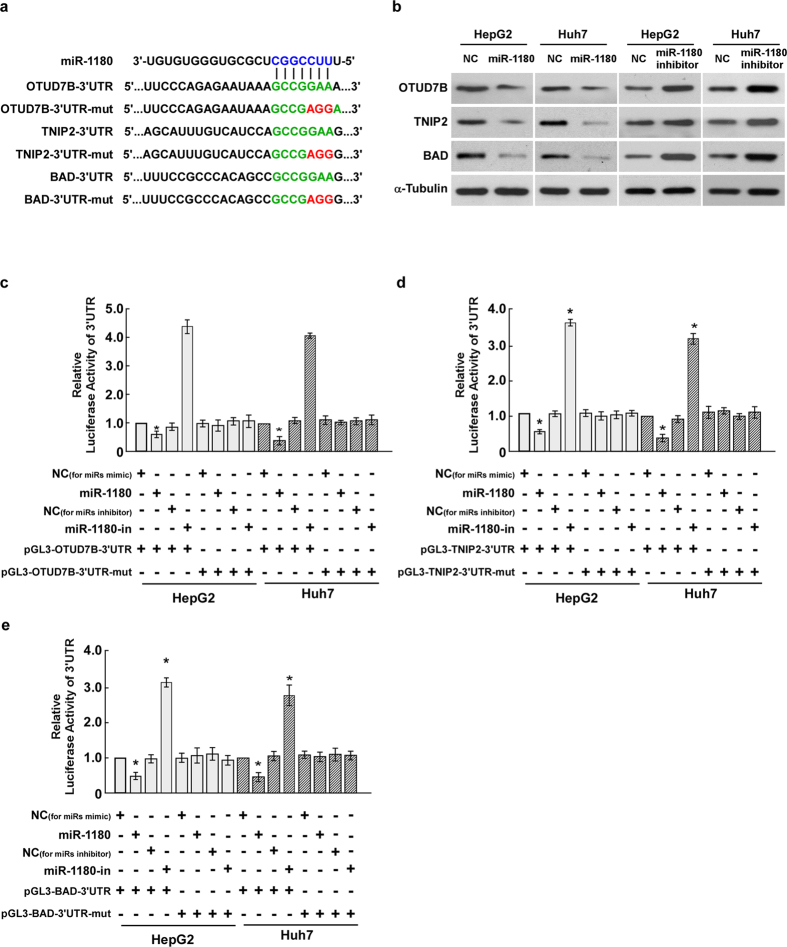
MiR-1180 directly targets the 3′-UTR of *OTUD7B*, *TNIP2*, and *BAD* mRNA. **(a)** Schematic representation of the mature miR-1180 sequence, the miR-1180 target site in the 3′-UTR of *OTUD7B*, *TNIP2*, and *BAD* mRNA, and a 3′-UTR mutant of *OTUD7B*, *TNIP2*, and *BAD* mRNA containing three altered nucleotides in the putative target site (*OTUD7B-*3′UTR-mut, *TNIP2-*3′UTR-mut, and *BAD*-3′UTR-mut). (**b)** The expression levels of OTUD7B, TNIP2, and BAD protein in HCC cells overexpressing miR-1180 or transfected with the miR-1180 inhibitor, compared with control cells (NC), by western blotting 48 h post-transfection; α-Tubulin is the loading control. (**c)** Luciferase assay of pGL3-*OTUD7B*-3′UTR or pGL3-*OTUD7B* -3′UTR-mut reporter cotransfected with miR-1180 mimic or miR-1180 inhibitor in indicated cells. (**d)** Luciferase assay of pGL3-*TNIP2*-3′UTR or pGL3-*TNIP2*-3′UTR-mut reporter cotransfected with miR-1180 mimic or miR-1180 inhibitor in indicated cells. (**e)** Luciferase assay of pGL3-*BAD*-3′UTR or pGL3-*BAD*-3′UTR-mut reporter cotransfected with miR-1180 mimic or miR-1180 inhibitor in indicated cells. Each bar represents the mean ± SD of three independent experiments. *P < 0.05.

To explore the molecular mechanism of miR-1180 in HCC cells, the publicly available algorithms (TargetScan) were used to predict the target(s) of miR-1180 in humans. The results showed that *OTUD7B*, *TNIP2* and *BAD*, which are closely correlated with cell survival and apoptosis progression in tumours, were three putative targets of miR-1180 (Fig. 5a). As predicted, western blotting revealed that the expression of OTUD7B, TNIP2 and BAD decreased in HepG2 and Huh7 cells overexpressing miR-1180, and expression increased in cells transfected with the miR-1180 inhibitor compared to negative controls (Fig. 5b). To further confirm the direct correlation between miR-1180 and these putative target genes, the *OTUD7B*-, *TNIP2*- and *BAD*-3′-UTR fragments, containing the miR-1180 binding site, were subcloned into a pGL3 luciferase reporter vector. Ectopic overexpression of miR-1180 decreased the activity of *OTUD7B*-, *TNIP2*- or *BAD*-3′-UTR-luciferase reporters and miR-1180 suppression increased their activity (Fig. 5c–e). However, when the *OTUD7B*-, *TNIP2*- or *BAD*-3′-UTR’s contained a mutated binding site (in the seed sequence), the luciferase activity was not affected by miR-1180 overexpression or suppression (Fig. 5c–e). Collectively, the results revealed that *OTUD7B*, *TNIP2* and *BAD* are direct targets of miR-1180, and are subsequently downregulated in HCC cells overexpressing miR-1180.

### Activation of NF-κB signaling pathway is essential for the apoptosis resistant function of miR-1180 in HCC cells

**Figure 6:**
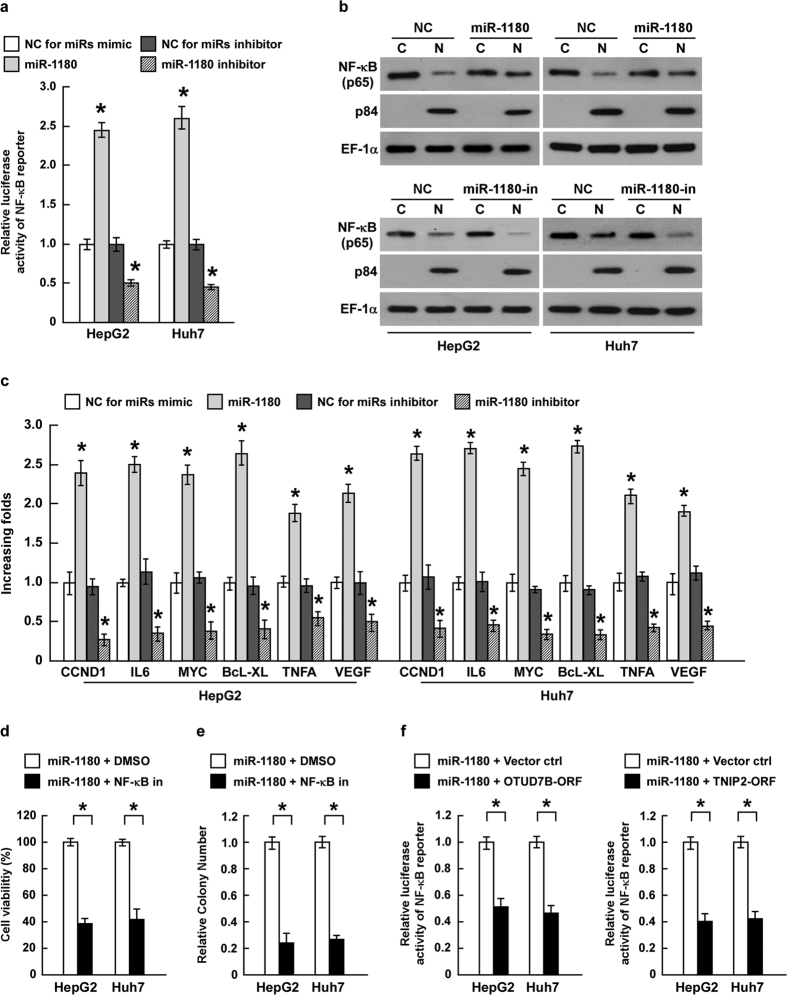
MiR-1180 activates the NF-κB signaling pathway. **(a)** NF-κB transcriptional activity measured by the luciferase assay in HepG2 and Huh7 cell lines in the presence of the miR-1180 mimic, the miR-1180 inhibitor, and a negative control (NC). **(b)** Western blotting analysis of NF-κB p65 expression in the cytoplasm (C) and nucleus (N) in the indicated cells. Nuclear protein p84 is a nuclear protein marker and EF-1α is a loading control. (**c)** The expression of NF-κB-targeted genes, *CCND1*, *IL6*, *MYC*, *BcL-XL*, *TNFA* and *VEGF*, measured by qRT-PCR; *GAPDH* is the control. (**d)** Cell viability in miR-1180 overexpressing cells treated with NF-κB inhibitor (NF-κ B-in) as measured by the MTS assay. (**e)** Quantifications of crystal violet stained cell colonies formed in miR-1180 overexpressing cells treated with NF-κB inhibitor (NF-κB in). (**f)** The relative NF-κB transcriptional activity measured by the luciferase assay in the indicated cells. Each bar represents the mean ± SD of three independent experiments. *P < 0.05.

As OTUD7B and TNIP2 act as inhibitors of the NF-κB signaling pathway^19^,^20^,^21^, we further examined the activity of NF-κB using a luciferase reporter assay. Ectopic miR-1180 overexpression promoted NF-κB transcriptional activity compared to controls (Fig. 6a). Moreover, the abundance of nuclear p65 significantly increased in the miR-1180-overexpressing cells and was reduced when miR-1180 was suppressed (Fig. 6b, Supplementary Figure 3a). Meanwhile, the effect of miR-1180 on p50 nuclear translocation is the similar to that on p65 (Supplementary Figure 3b). Our result was consistent with previous reports that activation of NF-κB leads to nuclear translocation of NF-κB (p65/p50) heterodimer to initiate target genes’ transcription^23^,^24^,^25^,^26^,^27^. The expression of NF-κB-targeted genes, including *CCND1*, *IL6*, *MYC*, *BcL-XL*, *TNFA* and *VEGF*, were examined by qRT-PCR. Overexpression of miR-1180 enhanced the transcription of all target genes, whereas miR-1180 suppression clearly depressed gene transcription in HepG2 and Huh7 cell lines (Fig. 6c). To further confirm miR-1180’s function on NF-κB signaling, cells transfected with miR-1180 were treated with an NF-κB inhibitor, JSH23, for 24 h. The MTS assay and colony formation assay both revealed that inhibition of NF-κB blocked the apoptosis resistant function of miR-1180 (Fig. 6d,e). Moreover, Overexpression of OTUD7B or TNIP2 indeed antagonizes miR-1180-induced NF-κB activation, which further confirmed our conclusion that miR-1180-regulating OTUD7B (or TNIP2)- NF-κB- signaling regulation mediates HCC resistance (Fig. 6f and Supplementary Figure 4). These experiments indicate that miR-1180 promotes the resistance to cisplatin-induce apoptosis in HCC cells through activation of the NF-κB signaling pathway.

### Clinical relevance of miR-1180-mediated OTUD7B and TNIP2 inhibition and NF-κB activation in HCC

**Figure 7:**
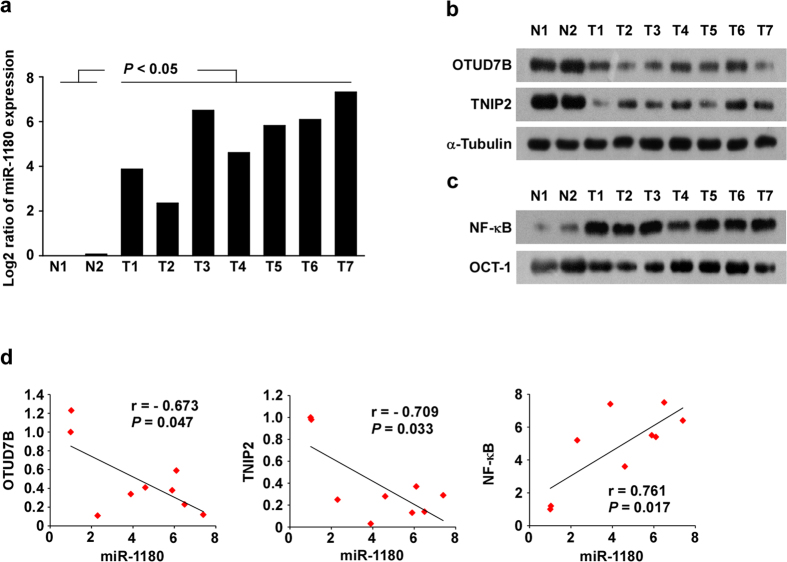
The expression of miR-1180, OTUD7B and TNIP2, and NF-κB activity in HCC tissues. (**a–c)** qRT-PCR analysis of miR-1180 (**a**), western blotting analysis (**b**) of OTUD7B and TNIP2 expression and EMSA analysis of NF-κB activity (**c**) in HCC tissues. Log2 value was used to show qRT-PCR results. (**d)** The correlation between miR-1180, OTUD7B and TNIP2 expression and NF-κB activity in HCC tissues. Error bars represent the mean ± SD from three independent experiments. *P < 0.05.

Finally, we examined whether miR-1180-mediated OTUD7B and TNIP2 inhibition, and NF-κB signaling activation, in HCC cells was clinically relevant. As shown in Fig. 7a,b, and Supplementary Figure 5, miR-1180 expression in seven freshly collected HCC samples was inversely correlated with expression of OTUD7B (r = −0.673, *P* = 0.047) and TNIP2 (r = −0.709, *P* = 0.033); but was positively correlated with NF-κB activation (r = 0.761, *P* = 0.017). Collectively, our results demonstrate that upregulation of miR-1180 activates NF-κB signaling via downregulation of OTUD7B and TNIP2, and thereby promotes the anti-apoptotic ability of HCC cells.

## Discussion

In the present study, we showed miR-1180 is upregulated in both HCC tissues and cell lines. Ectopic overexpression of miR-1180 was capable of increasing the HCC cell growth and resistance to cisplatin-induced cell apoptosis both *in vitro* and *in vivo*. On the other hand, miR-1180 suppression enhanced cisplatin-induced cell apoptosis. Moreover, we found that the direct targets of miR-1180 are *OTUD7B*, *TNIP2* and *BAD*, and their expression is downregulated by the microRNA in HCC cells. As OTUD7B and TNIP2 are common inhibitors of NF-κB signaling, we further examined this process and found that the NF-κB pathway was significantly activated by miR-1180, indicating its crucial role for apoptosis regulation might be through activation of NF-κB signaling in HCC cells.

Anticancer agents aimed to induce apoptosis are considered an effective therapeutic strategy to treat tumours. Importantly, the normal regulatory network of apoptosis is essential for apoptosis-induced therapy. Defects in apoptotic pathways (including mutations, downregulation, epigenetic silencing of death receptors, overexpression of FLIP, epigenetic silencing of Caspase, and alterations in the mitochondrial pathway) can result in apoptosis resistance and therapy failure^28^. Apoptosis resistance becomes more complicated as the tumour evolves, and multiple apoptotic genes and their regulators may play a part in the resistance progression. MiRNAs contribute to the cellular proliferation, differentiation and metastasis of various human malignancies, and have recently attracted considerable attention for their effects on apoptosis and drug resistance^29^,^30^,^31^,^32^,^33^,^34^,^35^. In particular, miR-21 has been reported to protect glioblastoma cells from the chemotherapeutic drug temozolomide-induced apoptosis by decreasing the Bax/Bcl-2 ratio and caspase-3 activity^16^. In addition, Li *et al.* found that the miR-106a was involved in the development of drug resistance of human ovarian cancer cells by targeting PDCD4, primarily through the death receptor-mediated pathway^36^. Similarly, in our study, we discover that miR-1180 is able to inhibit cisplatin-induced apoptosis of HCC cells. We also indicate a mechanism for miR-1180 induced drug resistance by downregulation of OTUD7B, TNIP2 and BAD, the direct targets of miR-1180 in HCC cells.

OTUD7B and TNIP2 are known inhibitors of the NF-κB signaling pathway; a pathway that has multiple functions during tumourigenicity and development, and targets genes involved in cell proliferation, anti-apoptosis, cell migration or invasion, and angiogenesis^37^,^38^,^39^,^40^,^41^. Previous studies by Hu *et al.* have reported that OTUD7B negatively regulates TRAF3 degradation by affecting its ubiquitination, thereby preventing aberrant activation of non-canonical NF-κB signaling^19^. Meanwhile, TNIP2 binds to the COOH-terminal domain of the zinc-finger protein A20 to inhibit NF-κB activation^20^. Additionally, miR-486 can disrupt multiple NF-κB-negative feedback loops, by downregulating CYLD, Cezanne, and multiple A20 regulators, including ITCH, TNIP-1, TNIP-2 and TNIP-3^21^. In the present study, we observe that miR-1180 is also capable of augmenting the activity of NF-κB signaling to increase the apoptosis resistance of HCC cells. This increased activity of NF-κB signaling by miR-1180 is suggested to occur via downregulation of OTUD7B and TNIP2. In addition, our study shows that miR-1180 downregulates the BAD protein, which selectively binds to anti-apoptotic molecules of the Bcl-2 family to mediates its pro-apoptotic functions^42^. On the other hand, survival signals lead to BAD phosphorylation, which results in their inactive localization in the cytoplasm^43^. As BAD is also downregulated by miR-1180, the mechanism may also contribute to cell survival in HCC. Collectively, our results are consistent with the expected outcomes of an activated NF-κB signaling pathway, and provide evidence that miR-1180 contributes to cisplatin-induced resistance in HCC by targeting this pathway.

## Conclusions

In summary, the present study demonstrates that the upregulation of miR-1180 contributes to the proliferation and cisplatin-resistance of HCC cells both *in vitro* and *in vivo*, by targeting and suppressing *OTUD7B*, *TNIP2* and *BAD*. Inhibition of OTUD7B and TNIP2 results in activation of NF-κB, suggesting the anti-apoptotic function of miR-1180 may occur through the NF-κB signaling pathway.

## Additional Information

**How to cite this article**: Tan, G. *et al.* MiR-1180 promotes apoptotic resistance to human hepatocellular carcinoma via activation of NF-κB signaling pathway. *Sci. Rep.* **6**, 22328; doi: 10.1038/srep22328 (2016).

## Supplementary Material

Supplementary Information
