# Supplementary material for: MiR-1180 promotes apoptotic resistance to human hepatocellular carcinoma via activation of NF-κB signaling pathway
Source: Sci Rep. 2016 Mar 1;6:22328. doi: 10.1038/srep22328 (PMC4772113; doi:10.1038/srep22328)
Supplement: Supplementary Information [file srep22328-s1.pdf]

MiR-1180 promotes apoptotic resistance to human hepatocellular carcinoma via activation of NF-κB signaling pathway

Guosheng Tan[1]*. Linwei Wu[2]*, Jinfu Tan[3], Bing Zhang[4], William Chi-shing Tai[5,6], Shiqiu Xiong[7], Wei Chen[1], Jianyong Yang[1§], Heping Li[1,8§]

1 Department of Interventional Radiology, the First Affiliated Hospital of Sun Yat-sen University, Guangzhou 510080, P.R.China.

2 Organ Transplantation Center, the First Affiliated Hospital, Sun Yat-Sen University, Guangzhou, China

3 Department of Anorectal & Hernial Surgery, the First Affiliated Hospital of Sun Yat-sen University, Guangzhou 510080, P.R.China.

4 Department of Nuclear Medicine, the First Affiliated Hospital of Sun Yat-sen University, Guangzhou 510080, P.R.China.

5 Centre for Cancer and Inflammation Research, School of Chinese Medicine, Hong Kong Baptist University. Kowloon Tong, Hong Kong, S.A.R., P.R.China.

6 Institute of Integrated Bioinformedicine and Translational Science, Hong Kong Baptist University, Shenzhen Research Institute and Continuing Education, Shenzhen 518000, P.R.China.

7 Department of Biochemistry, University of Leicester, Leicester, UK.

8 Department of Medical Oncology, the First Affiliated Hospital of Sun Yat-sen University. Guangzhou 510080, P.R.China.

*These authors contributed equally to this work

§Corresponding author: Tel/Fax: +86 20 37621096, E-mail: **drliheping@163.com** (Heping Li)

Supplementary information

Supplementary Figure 1

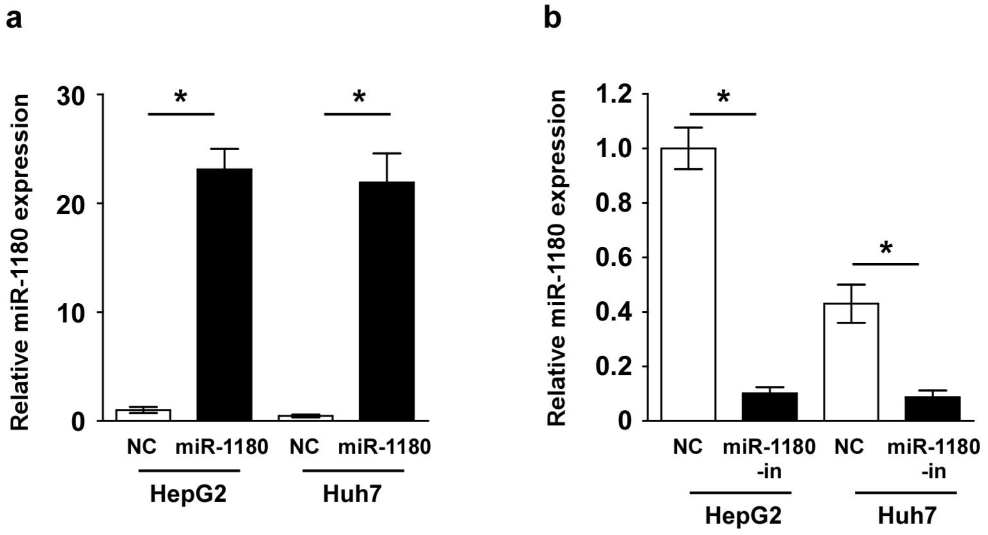

Supplementary Figure 2

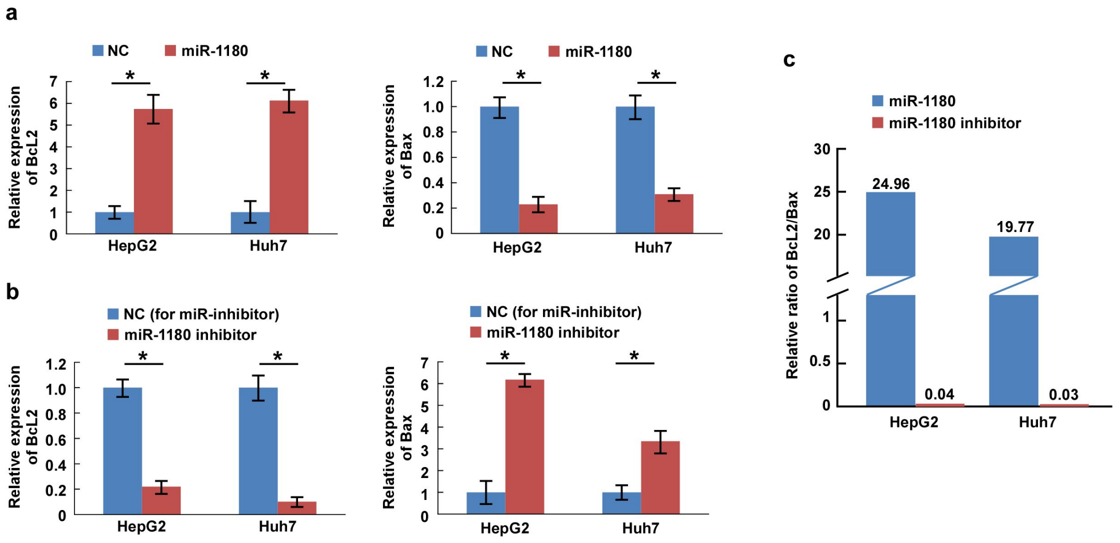

Supplementary Figure 3

a

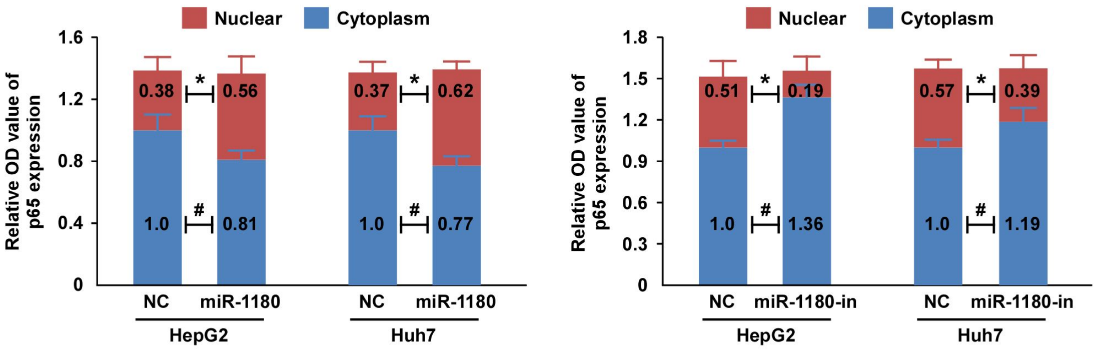

b

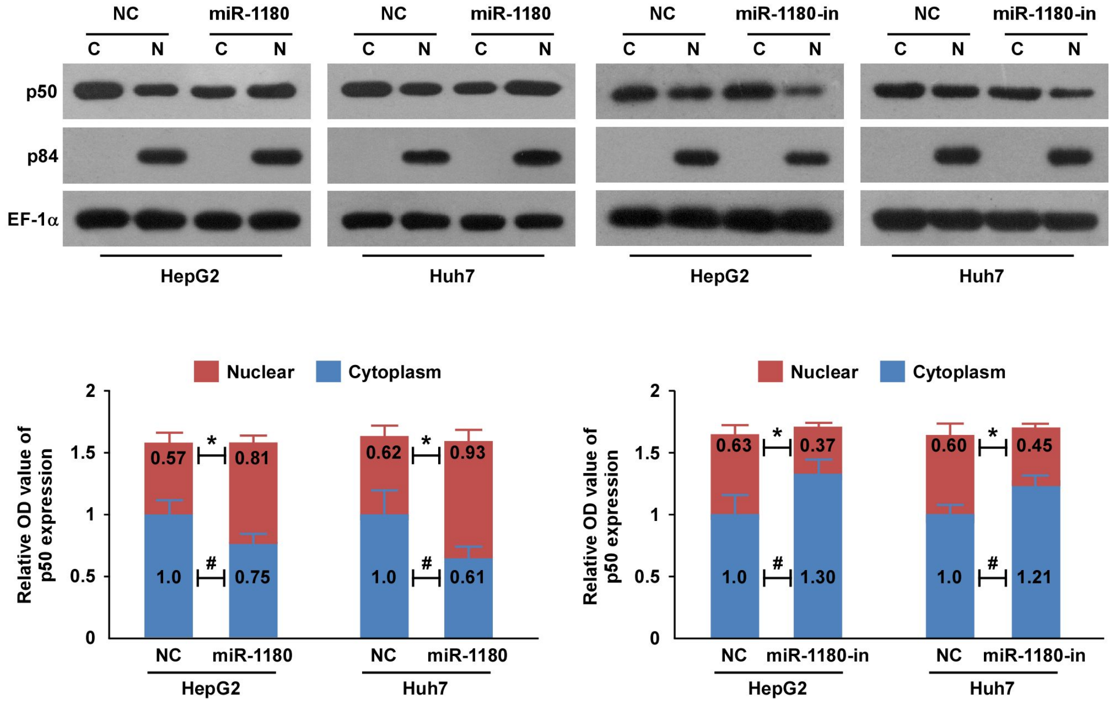

Supplementary Figure 4

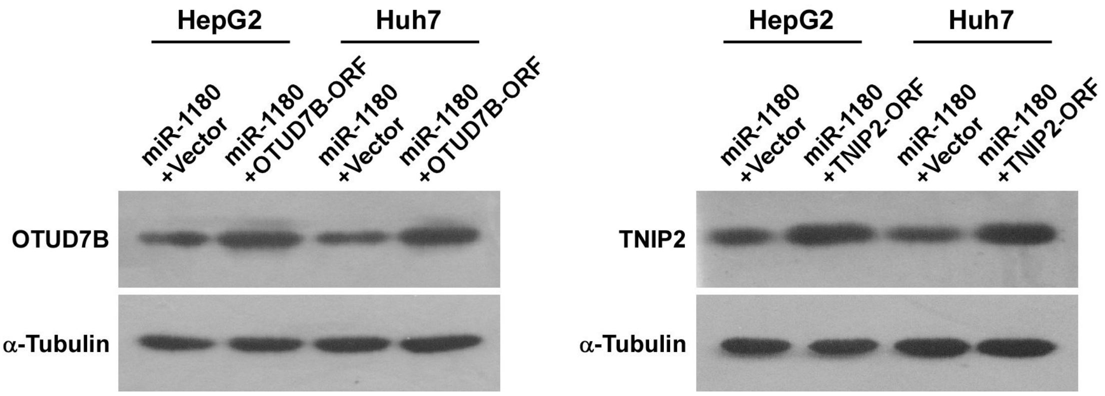

Supplementary Figure 5

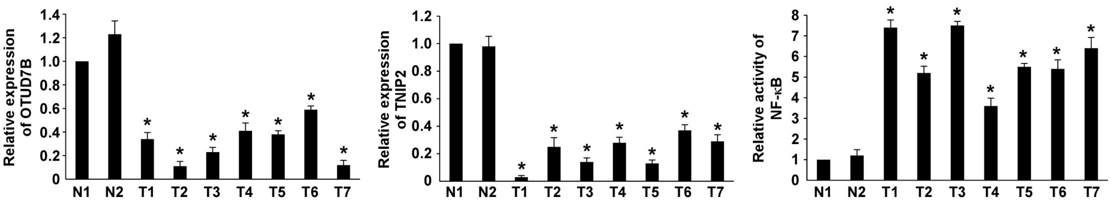

Supplementary Figure for reviewer and editor

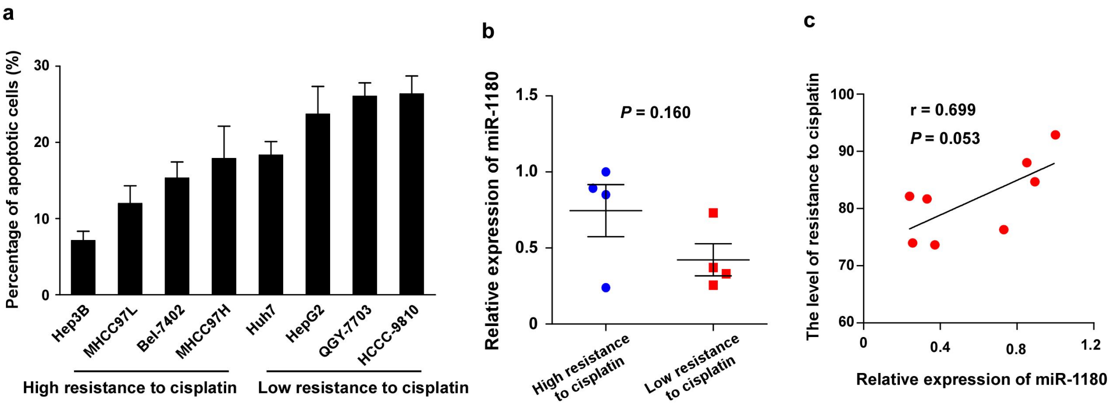

a. The percentages of apoptotic cells in HCC cell lines, determined by flow cytometry analysis. b. The relative expression of miR-1180 in group of high or low resistance to cisplatin. c. The correlation between the expression of miR-1180 and the level of resistance to cisplatin.

Supplementary figure legends

Supplementary Figure 1: The expression of miR-1180 in HepG2 and Huh7 overexpressing (a) or silencing miR-1180 (b). The expression level was normalized with miR-1180 expression of HepG2-NC. Each bar represents the mean ± SD of three independent experiments. **P* <0.05.

Supplementary Figure 2: miR-1180 promotes anti-apoptosis ability of HCC cells. a and **b.** The relative expression of BcL-2 and Bax in indicated cells, determined by quantification of western blotting assay. The quantification of western blotting bands is performed by OD value determined by Quantity One software. **c.** The relative ratio of BcL-2 and Bax in indicated cells. Each bar represents the mean ± SD of three independent experiments. **P* <0.05.

Supplementary Figure 3: MiR-1180 activates NF-κB signaling pathway. a. The quantification of NF-κB p65 expression in the cytoplasma (C) and nuclear (N) by western blotting analysis in indicated cells. The quantification of western blotting bands is performed by OD value determined by Quantity One software. **b.** Western blotting analysis of p50 expression (upper) and quantification (lower) in the cytoplasma (C) and nuclear (N) in indicated cells. Nuclear protein p84 was detected as a nuclear protein marker and EF-1α as a loading control. The quantification of western blotting bands is performed by OD value determined by Quantity One software. Each bar represents the mean ± SD of three independent experiments. *P <0.05.

Supplementary Figure 4 - OTUD7B and TNIP2 are essential for miR-1180 mediated NF-κB signaling activation. Western blotting analysis of OTUD7B and TNIP2 in indicated cells.

Supplementary Figure 5: The relative expression of OTUD7B and TNIP2, and NF-κB activity in HCC tissues. The quantification of western blotting bands and EMSA bands are performed by OD value determined by Quantity One software. Each bar represents the mean ± SD of three independent experiments. *P <0.05.
